# Ellagic acid improved arrhythmias induced by CaCL_2_ in the rat stress model

**Published:** 2015

**Authors:** Mahin Dianat, Negin Amini, Mohammad Badavi, Yaghoub Farbood

**Affiliations:** 1 *Physiology Research Center and Department of Physiology, Faculty of Medicine, Ahvaz Jundishapur University of Medical Sciences, Ahvaz, Iran*

**Keywords:** *Ellagic acid*, *Arrhythmia*, *Inotropic*, *Chronotropic*, *Stress*, *Rat*

## Abstract

**Objective:**

In ventricular arrhythmias, due to their free radical scavenging action, antioxidant agents are usually used in the treatment of cardiovascular disease. Since stress is considered as risk factor for increased mortality by causing malignant arrhythmias, the study was designed to evaluate the cardioprotective effects of ellagic acid (EA) on CaCl_2_-induced arrhythmias in rat stress model.

**Materials and Methods:**

Male Sprague-Dawley rats (200-250 g) were divided into four groups: Group I: Control rats (2 ml of saline by gavage), Group II: Rats treated with EA (15 mg/kg, gavage), Group III: stress group, Group IV: received EA plus stress. Stress was applied in a restrainer box (6 hour/day, 21 days). After induction of anesthesia, lead II electrocardiogram was recorded for calculating heart rate and QRS complex. The arrhythmia was produced by injection of CaCl_2_ solution (140 mg/kg, iv) and incidences of Ventricular fibrillation, Ventricular premature beats and Ventricular tachycardia were recorded. Results were analyzed by using one-way ANOVA and Fisher`s exact test. p<0.05 was considered as significant level.

**Results:**

The results showed a positive inotropic effect and negative chronotropic effect for the EA group in comparison with the control group. Incidence rates (%) of premature beats, ventricular fibrillation and ventricular tachycardia in stress group and all the arrhythmia parameters decreased in groups which received EA.

**Conclusions:**

By decreasing the incidence rates of premature beats, fibrillation and ventricular tachycardia in groups which received EA, ellagic acid probably acted as an anti-arrhythmic agent which showed to have aprotective functionin heart.

## Introduction

Sudden cardiac death is the leading cause of deaths worldwide (Chugh et al., 2008 ▶). One cause of sudden cardiac death is ventricular arrhythmias (Chugh et al., 2008 ▶) that could be due to disturbances of Ca^2+^ signaling (Belevych et al., 2009 ▶), ionic imbalances during myocardial ischemia (Rubart and Zipes, 2005 ▶) and excessive activation of sympathetic system (Rubart and Zipes, 2005 ▶). Studies have demonstrated that CaCl_2_ initiates arrhythmias directly and indirectly by activation of sympathetic nervous system (Grumbach et al., 1954 ▶; Malinow et al., 1953 ▶). 

There are significant evidence indicating that free radicals could be an important mediator of ventricular tachycardia and ventricular fibrillation (Beresewicz and Horackova, 1991 ▶; Gelvan et al., 1991 ▶). Furthermore, oxidative stress is a substantial factor in cardiovascular disease that can trigger ventricular tachycardia and ventricular fibrillation (Karagueuzian et al., 2013 ▶). Oxidative stress is defined as excessive production of reactive oxygen species (ROS) (Chen and Keaney, 2012 ▶). ROS include free radicals such as superoxide, peroxyl and non-radical species (Chen and Keaney, 2012 ▶). 

Meerson’s study has shown that stress is one of the risk factors of increase in mortality and can induce myocardial ischemia or arrhythmias (Meerson, 1994 ▶). Cardiovascular disease and cardiac arrhythmias are proved to be related to the stress (Rozanski et al., 1999 ▶). Animal studies have shown that stress can lead to hormonal disturbances such as increase in corticosterone and norepinephrine (Irwin et al., 1986 ▶; Marin et al., 2007 ▶), Histamine and serotonin disturbances (Carnevali et al., 2012 ▶; He et al., 2009 ▶). Experimental studies have shown that chronic stress and increase in oxidative stress biomarkers including malondialdehyde (MDA), xanthine oxidase activity (XOD) and lipid peroxidation as products of oxidative stress might initiate cell damages in response to chronic stress exposure (Kaushik and Kaur, 2003 ▶). 

Other studies have reported that chemical drugs used in the treatment of arrhythmias have considerable side effects (Khori V, 2007 ▶). 

Numerous studies have reported that dietary polyphenols have an important role in the prevention of arrhythmias (Kannan and Quine, 2013 ▶) and hypertension disease (Furuuchi et al., 2012 ▶). Ellagic acid (EA) is a polyphenol compound that has attracted the attention of many researchers as an agent that protects cells against oxidative stress (Kannan and Quine, 2011 ▶). Ellagic acid exists in vegetables, nuts and fruits such as pomegranate, black berry, grapes, strawberries and walnuts (Daniel et al., 1989 ▶; Elfalleh et al., 2011 ▶). Previous studies on EA have shown that it has antioxidant (Devipriya et al., 2007 ▶), antihyperlipidaemic and antihypertensive properties (Kannan and Quine, 2011 ▶; Kannan and Quine, 2013 ▶).

Since the effect of EA has not been yet evaluated by CaCl_2_ – induced arrhythmias and side effect of stress on health has been expressed, our study was designed to evaluate the chronic stress protocols, chronotropic and inotropic properties of EA and arrhythmia induced by CaCl_2 _in rats.

## Material and Methods

**Animals and grouping**

Thirty-two male Sprague Dawley rats (weighing 200- 250 g) were purchased from Animal reproduction center. The animals were housed in groups of four per cage under conditions of temperature (22 °C) humidity (50%) and photoperiod (12 hours on, 12 hours off). The rats were fed on a standard pellet diet and drinking water. 

The experiment was approved by the Animal Ethics Committee of Ahvaz Jundishapur University of Medical Sciences (No. ajums. REC.1392.280).

The rats were divided randomly into four groups of eight rats each. Group I: normal control rats receiving 2 ml of saline orally by gavage daily for a period of 10 days, Group II: normal rats treated with EA (15 mg/kg) in 2 ml of saline orally by gavage daily for a period of 10 days (Kannan and Quine, 2011 ▶), Group III: Rats placed in restrainter box (6 hour/day for 21 days)(McLaughlin et al., 2007 ▶), Group IV: Rats pretreated EA (15 mg/kg, gavage, 10 days) plus stress (6 hour/day for 21 days). 

**Chronic stress protocol**

In this study, the stress was applied in a restrainer box and was applied between from 8:00 to 14:00 h, 6 hour/day for 21 successive days (McLaughlin et al., 2007 ▶). 

**Surgical method**

On the first day and by the end of 21-day period, all rats were anesthetized with a mixture of ketamine (50 mg/kg) andxylazine (10 mg/kg) by intraperitoneal injection (Dianat et al., 2013 ▶). Fifteen minutes after anesthesia, standard bipolar limb lead II was recorded for record the electrocardiogram (ECG). Lead II ECG was recorded by Bio-Amp and monitored by a Power Lab system (AD-Instruments, Australia). At the end of the 21st day in all groups, after anesthesia, standard bipolar limb lead II ECG was recorded to evaluate chronotropic and inotropic properties. In order to investigate arrhythmia, a longitudinal slot in the groin was created and the femoral vein was exposed. Then the femoral vein was cannulated by a polyethylene catheter for injection of a 2.5% CaCl_2 _solution (140 mg/kg) (Dianat et al., 2013 ▶). After injection of CaCl_2_, incidence rates of premature ventricular beats (PVB), ventricular fibrillation (VF) and ventricular tachycardia (VT) were calculated.

**Statistical analysis**

Between groups comparisons were analyzed by one-way ANOVA while for within group comparisons Paired t-test was used. Fisher`s exact test was also employed to evaluate the arrhythmias data. The values p<0.05 were considered significant level.

## Results

**Figure 1:**
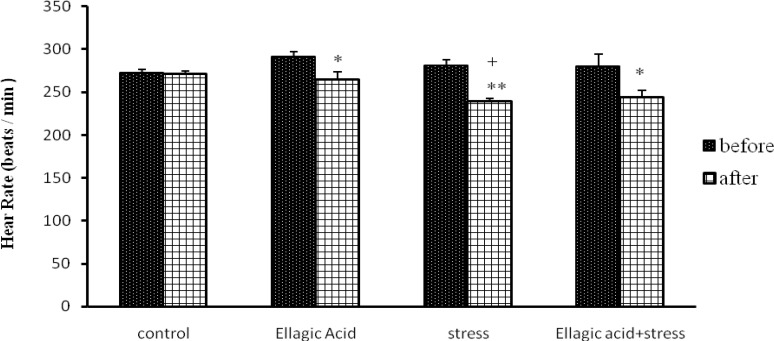
Comparison of Heart rate in different groups [(control), (Ellagic Acid 15 mg/kg), (stress, 21 days), (Ellagic Acid + Stress)]. *p<0.05, **p<0.01 Significant differences before and after treatment and +p<0.05 Significant differences with control group (n=8, Mean±SEM, Paired t-Test or One-way ANOVA followed by LSD).

**Figure 2:**
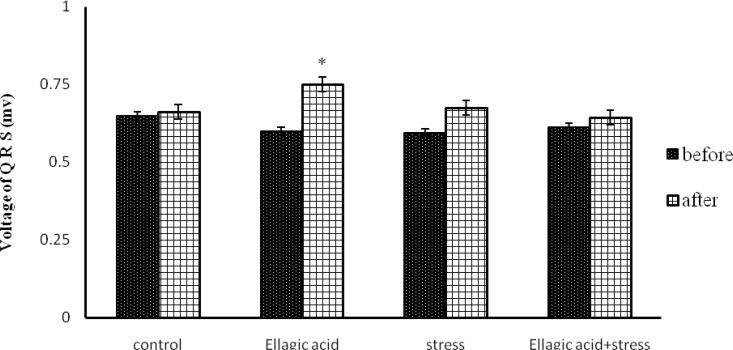
Comparison of Voltage of QRS in different groups [(control), (Ellagic Acid 15 mg/kg), (stress, 21 days), (Ellagic Acid + Stress)]. *p<0.05 Significant differences before and after treatment. (n=8, Mean±SEM, Paired t-Test or One-way ANOVA followed by LSD).

The Rats that received EA (15 mg/kg) for 10 days, showed a significant decrease in heart rate (p<0.05) (Figure 1) and their QRS complex voltage significantly increased (p<0.05) (Figure. 2).

**Figure 3:**
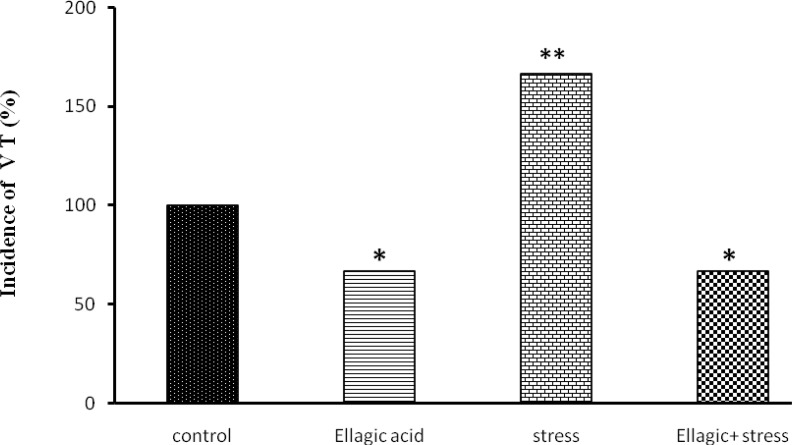
Evaluation of ventricular Tachycardia after Chemical arrhythmias inducing by intravenous CaCl_2_ injection (140 mg/kg) in different rats groups [(control), (Ellagic Acid 15 mg/kg), (stress, 21 days), (Ellagic Acid + Stress)]. *p<0.05, **p<0.01 Significant differences with control group (n=8, Fisher`s exact test).

And heart rate significantly reduced in chronic stress group (p<0.01) (Fig. 1) and their QRS complex voltage was not significant compared to that of the normal control group (Fig. 2). Rats exposed to chronic stress and receiving EA, showed a significant decrease in heart rate (p<0.05) while their QRS complex voltage was not significant (Fig. 1, 2). 

**Figure 4:**
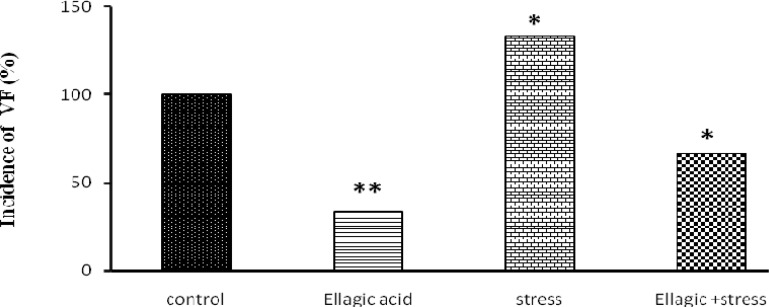
Evaluation of ventricular fibrillation after Chemical arrhythmias inducing by intravenous CaCl_2_ injection (140 mg/kg) in different rats groups [(control), (Ellagic Acid 15 mg/kg), (stress, 21 days), (Ellagic Acid + Stress)]. *p<0.05, **p<0.01 Significant differences with control group (n=8, Fisher`s exact test).

**Figure 5:**
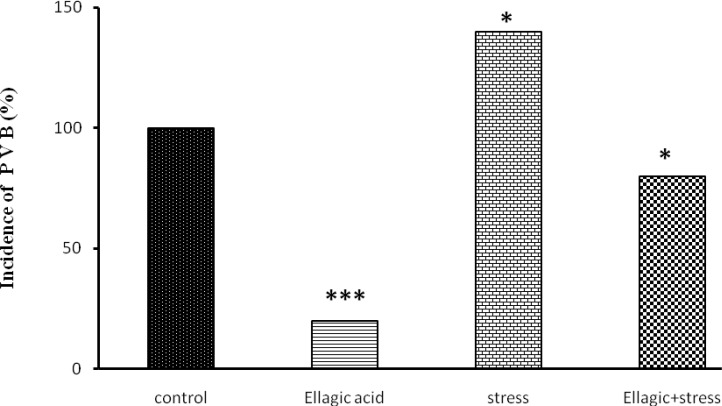
Evaluation of premature ventricular beats after Chemical arrhythmias inducing by intravenous CaCl_2_ injection (140 mg/kg) in different rats groups [(control), (Ellagic Acid 15 mg/kg), (Stress), (Ellagic Acid + Stress)]. *p<0.05, ***p<0.001. Significant differences with control group (n=8, Fisher`s exact test).

Rats receiving EA (15 mg/kg), showed a significant decrease in percentages of incidence of ventricular tachycardia (p<0.05) (Fig. 3), Ventricular fibrillation (p<0.01) (Fig. 4) and Ventricular premature beats (p<0.001) (Fig. 5) Rats exposed to chronic stress for 21 days (6 hours per day), showed a significant increase in percentages of incidence of ventricular tachycardia (p<0.01), Ventricular fibrillation (p<0.01) and Ventricular premature beats (p<0.01). Finally, rats exposed to chronic stress and receiving EA (15 mg/kg), showed a significant decrease in percentage of incidence of ventricular tachycardia (p<0.05), Ventricular fibrillation (p<0.05) and Ventricular premature beats (p<0.05) (Fig. 3-5).

## Discussion

In this experimental study, in rats that received EA, decreased heart rate and increased QRS complex voltage were documented. 

Other studies have shown that by increasing calcium absorption in Sarcoplasmic reticulum and increasing Ca^2+^-ATPase Pump activity, EA inducesa positiveinotropiceffect (Antipenko et al., 1999 ▶). EA has been shown to have free radical scavenging activity (Devipriya et al., 2007 ▶), it is also inhibits numbers of factors in reduction QRS complex voltage and lead to positiveinotropic of effects in heart tissue (Antipenko et al., 1999 ▶). A recent study showed that administration of EA reduces heart rate, systolic and diastolic blood pressure in isoproterenol induced myocardial infarction rats, and also an increase in superoxide dismutase, glutathione peroxidase, and catalase was observed (Kannan and Quine, 2011 ▶). It has been shown that polyphenol compounds bond with beta-adrenergic receptors and prevent heart rate from increasing (Zhu et al., 1997 ▶). The decrease in hear rate in this study could be due to the binding of EA with beta-adrenergic receptors. 

The present study showed that heart rate decreases in stress group (p<0.01) but the voltage of complex does not changed. It seems that when rats are exposed to stress, they adjust to this condition. The inotropic effect cannot be explained and requires further investigation. The study indicated that there are stress-limiting systems for the protection of organism; one example of such a system is the documented activity of parasympathetic system that leads to bradycardia (Meerson, 1994 ▶). 

In the present study, the rats treated with EA (15 mg/kg) showed decreased percentages of incidence ofVT, VF, and VPB. It has been already shown that oral pretreatment of EA attenuate electrocardiological parameter changes such as Q wave pathological and ST- segment elevation in induced-isoproterenol myocardial infarction rats (Kannan and Quine, 2011 ▶). Previous studies have shown that the consumption of polyphenol compounds such as EA reduces the levels of VLDL, LDL, triglyceride that are one of causes of cardiac arrhythmia (Kannan and Quine, 2013 ▶; Liu et al., 2006 ▶). Studies have also reported that EA has an inhibitory effect on HMGCoA reductase (rate limiting enzyme in cholesterol biosynthesis) (Kannan and Quine, 2013 ▶). An experimental study indicated that statins had antiarrhythmic properties whichare decreasing the incidence ofarrhythmias in coronary artery disease (Tamargo et al., 2007 ▶). Also, another study has shown that EA decreases the levels of thiobarbituric acid reactive substances and lipid hydroperoxides in induced- isoproterenol myocardial infarction rats (Kannan and Quine, 2013 ▶) that this may be due to the protective effect of EA against cardiac arrhythmias. However, a detailed study on EA as antidysrhythmic agent and its mechanism in regulating arrhythmias is needed.

A significant increase in ventricular tachycardia (p<0.01), Ventricular fibrillation (p<0.01) and Ventricular premature beats (p<0.001) was shown in our study. Studies of mechanism of stress damage to heart have indicated that its main component is increased catecholamines hormones (Irwin et al., 1986 ▶). A recent study suggested that histamine increases in response to stress (He et al., 2009 ▶). Previous studies have shown that peripheral infusion of histamine increases ventricular arrhythmias, like ventricular tachycardia, and injection of histamine H_1 _antagonists reduces ventricular tachycardia and ventricular fibrillation induced by myocardial ischemia (He et al., 2012 ▶). It has been reported that oxidative stress biomarkers such as xanthine oxidase, malondialdehyde (MDA) and lipid peroxidation increase in chronic stress model, and also the activity of superoxide dismutase and glutathione is reduced, which was ledto damage tothe cells in response to stress (Kaushik and Kaur, 2003 ▶). It is possible that the increase in ventricular arrhythmias happens due to the increased activity of oxidative stress biomarkers. 

In our study, rats which received EA plus stress, had significantly a decreased heart rate (P<0.05) but no significant differences were observed in QRS complex voltage. It seems that EA failed to apply its inotropic properties and stress eliminates the effect of its provision. In the evaluation of stress with ellagic acid, it was observed that percentages of incidence of ventricular tachycardia (p<0.05), Ventricular fibrillation (p<0.05) and Ventricular premature beats (p<0.05) significantly decreased. An experimental study has demonstrated that dietary polyphenols ameliorated cardiac damage in myocardial infarction is due to free radical scavenging and strong antioxidant effects (Kumaran and Prince, 2010 ▶). Our findings are in agreement with what previous studies suggested.

In summary, based on the results of this study, we conclude that oral pretreatment of EA has a cardio-protective effect. Also, incidence rate of PVB, VF and VT in groups which received EA decreased; therefore, this substance is suggested as an antiarrhythmic factor which shows a protective function in heart. Therefore, EA can be used as a protective factor in the prevention of cardiovascular disease.

## Conflict of interest

There is no conflict of interest.
